# Sodium-glucose cotransporter 2 inhibitors, inflammation, and heart failure: a two-sample Mendelian randomization study

**DOI:** 10.1186/s12933-024-02210-5

**Published:** 2024-04-02

**Authors:** Wenqin Guo, Lingyue Zhao, Weichao Huang, Jing Chen, Tingting Zhong, Shaodi Yan, Wei Hu, Fanfang Zeng, Changnong Peng, Hongbing Yan

**Affiliations:** 1 Fuwai Hospital Chinese Academy of Medical Sciences, Shenzhen, China; 2 Huazhong University of Science and Technology Union Shenzhen Hospital, Shenzhen, China; 3 National Center for Cardiovascular Diseases, Fuwai Hospital, Peking Union Medical College and Chinese Academy of Medical Sciences, Beijing, China

**Keywords:** Heart failure, Sodium-glucose cotransporter 2 inhibition, Inflammatory biomarkers, Mendelian randomization

## Abstract

**Background:**

Sodium-glucose cotransporter 2 (SGLT-2) inhibitors are increasingly recognized for their role in reducing the risk and improving the prognosis of heart failure (HF). However, the precise mechanisms involved remain to be fully delineated. Evidence points to their potential anti-inflammatory pathway in mitigating the risk of HF.

**Methods:**

A two-sample, two-step Mendelian Randomization (MR) approach was employed to assess the correlation between SGLT-2 inhibition and HF, along with the mediating effects of inflammatory biomarkers in this relationship. MR is an analytical methodology that leverages single nucleotide polymorphisms as instrumental variables to infer potential causal inferences between exposures and outcomes within observational data frameworks. Genetic variants correlated with the expression of the SLC5A2 gene and glycated hemoglobin levels (HbA1c) were selected using datasets from the Genotype-Tissue Expression project and the eQTLGen consortium. The Genome-wide association study (GWAS) data for 92 inflammatory biomarkers were obtained from two datasets, which included 14,824 and 575,531 individuals of European ancestry, respectively. GWAS data for HF was derived from a meta-analysis that combined 26 cohorts, including 47,309 HF cases and 930,014 controls. Odds ratios (ORs) and 95% confidence interval (CI) for HF were calculated per 1 unit change of HbA1c.

**Results:**

Genetically predicted SGLT-2 inhibition was associated with a reduced risk of HF (OR 0.42 [95% CI 0.30–0.59], P < 0.0001). Of the 92 inflammatory biomarkers studied, two inflammatory biomarkers (C-X-C motif chemokine ligand 10 [CXCL10] and leukemia inhibitory factor) were associated with both SGLT-2 inhibition and HF. Multivariable MR analysis revealed that CXCL10 was the primary inflammatory cytokine related to HF (MIP = 0.861, MACE = 0.224, FDR-adjusted P = 0.0844). The effect of SGLT-2 inhibition on HF was mediated by CXCL10 by 17.85% of the total effect (95% CI [3.03%–32.68%], P = 0.0183).

**Conclusions:**

This study provides genetic evidence supporting the anti-inflammatory effects of SGLT-2 inhibitors and their beneficial impact in reducing the risk of HF. CXCL10 emerged as a potential mediator, offering a novel intervention pathway for HF treatment.

**Graphical Abstract:**

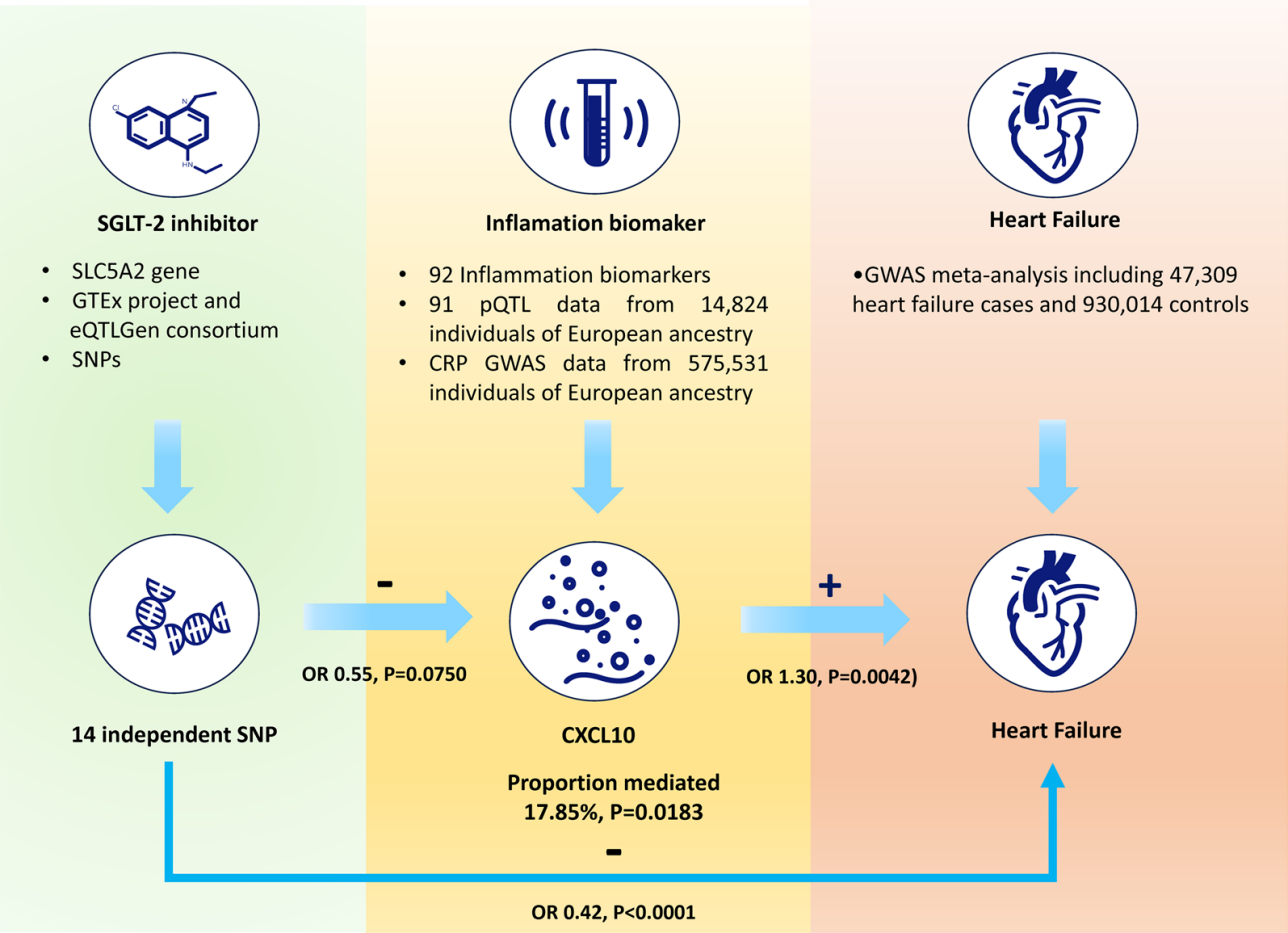

**Supplementary Information:**

The online version contains supplementary material available at 10.1186/s12933-024-02210-5.

## Introduction

Sodium-glucose cotransporter 2 (SGLT-2) inhibitors represent a novel class of antihyperglycemic drugs that act by inhibiting SGLT-2 in the renal tubules, thereby reducing the reabsorption of filtered glucose and enhancing glycosuria effects [1]. Further research has clarified the role of SGLT-2 inhibitors beyond blood glucose reduction, especially highlighting their potential in the management of heart failure (HF). Empirical data supports that SGLT-2 inhibitors significantly reduce the risk of hospitalization for HF treatment in type 2 diabetes patients without a history of HF [2]; furthermore, SGLT-2 inhibitors have significantly reduced the composite endpoint of cardiovascular death or hospital readmissions due to worsening HF in patients with HF [3]. However, the precise mechanisms through which SGLT-2 inhibitors reduce the risk of HF and improve prognosis have not been fully elucidated.

Emerging studies have posited that the prognostic severity of HF correlates with elevated levels of novel inflammatory markers, which have been shown to outperform established HF biomarkers [4]. Cell-based and animal model research further corroborates the anti-inflammatory properties of SGLT-2 inhibitors [5, 6]. This paves the way for the hypothesis that SGLT-2 inhibitors may beneficially impact HF by modulating inflammatory responses.

Mendelian Randomization (MR) is an analytical methodology that leverages single nucleotide polymorphisms (SNPs) as instrumental variables to infer potential causal inferences between exposures and outcomes within observational data frameworks [7]. This design paradigm capitalizes on the stochastic distribution of alleles during gametogenesis to emulate the methodological rigor of randomized intervention allocations typical of randomized controlled trials.

In line with these premises, we posit that inflammatory biomarkers may mediate the influence of SGLT-2 inhibitors on HF risk. Our study initially engages in a two-sample MR assessment to interrogate the association between SGLT-2 inhibition and HF. This is followed by a sophisticated two-step MR analysis employing an array of 92 inflammatory biomarkers to unravel the potential pathways through which SGLT-2 inhibitors exert their influence on HF risk, thereby augmenting our understanding of the inflammatory underpinnings related to the cardiac benefit of SGLT-2 inhibitors.

## Methods

**Fig. 1:**
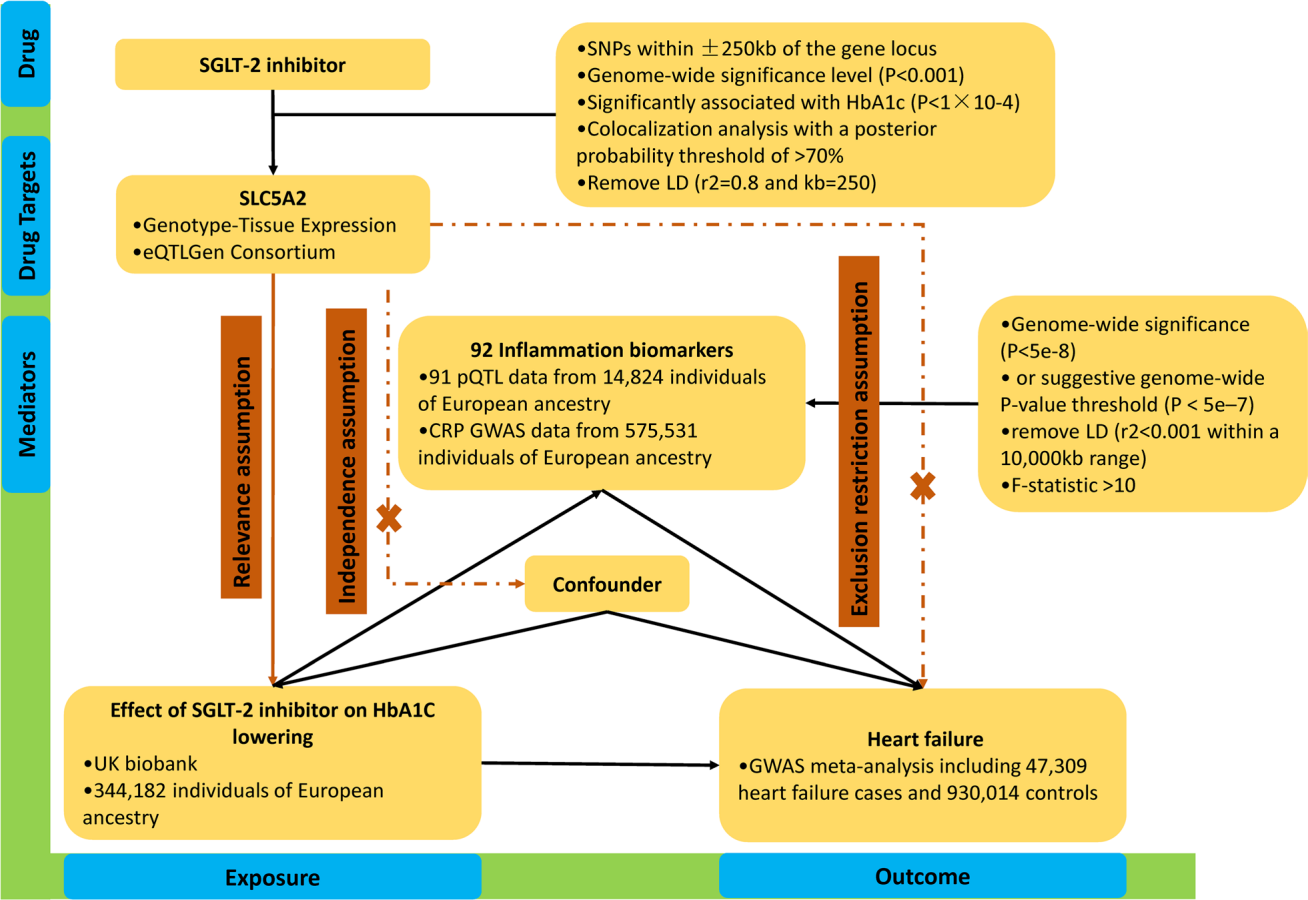
The flowchart of two-sample and two-step mendelian randomization evaluating the effects of inflammation biomarkers in mediating the effect of SGLT-2 inhibition on heart failure. *HbA1c* glycated hemoglobin, *GWAS* genome-wide association study, *pQTL* protein quantitative trait loci *SNP* single nucleotide polymorphism, *LD* linkage disequilibrium, *CRP* C-reactive protein, *SGLT-2* sodium-glucose Cotransporter-2

This study adhered to the guidelines of the Strengthening the Reporting of Observational Studies in Epidemiology using Mendelian Randomization (STROBE-MR) [8]. A two-sample MR design was employed to ensure the validity of causal effects (Fig. 1). MR analysis relies on three core assumptions: (1) the relevance assumption, which states that genetic variation is robustly associated with exposure; (2) the independence assumption, which states that genetic variation is independent of confounding factors; and (3) the exclusion restriction assumption, which states that genetic variation affects the outcome only through the exposure (Fig. 1).

## Genetic instruments for SGLT-2 inhibitors

We selected genetic variants for SGLT-2 inhibitors through the following steps (Fig. 1), which were described in the previous study [9]. First, we used genetic loci associated with mRNA expression to create instrumental variables representing the treatment effects of SGLT-2 inhibitors. We selected genetic variants associated with SGLT-2 mRNA expression levels using publicly available datasets from the Genotype-Tissue Expression (GTEx) project [10] and the eQTLGen consortium [11], and filtered for SNPs within ± 250 kb of the gene locus that were significantly associated with the corresponding trait at a genome-wide significance level (P < 0.001). Third, considering the glucose-lowering effect of SGLT-2 inhibitors, we estimated the association of each SGLT-2 variant with glycated hemoglobin (HbA1c) levels, an indicator of the glucose-lowering effect, and selected variants significantly associated with HbA1c (P < 1 × 10–4). The genome-wide association study (GWAS) data for HbA1c came from the UK Biobank and included 344,182 individuals of European ancestry (Additional file 1: Table S1). Finally, we performed colocalization analysis for SGLT-2 and HbA1c, using a posterior probability threshold of > 70% as evidence of colocalization, and performed clumping analysis with PLINK to remove SNPs in strong linkage disequilibrium (r2 = 0.8 and kb = 250) based on the 1000 Genomes Project reference panel of individuals of European ancestry.

## Genetic instruments for inflammatory biomarkers

The data for inflammatory biomarkers were obtained from the study by Zhao et al. (Additional file 1: Table S1) [12]. This study conducted a comprehensive assessment of genetic effects on inflammation-related proteins through a genome-wide study of protein quantitative trait loci (pQTL). The study included 14,824 participants and measured 91 plasma proteins using the Olink panel. Additionally, C-reactive protein (CRP), a commonly studied inflammatory marker, was not included in the study by Peters et al. [12] We used the summary genetic information of European ancestry for CRP-related genetic variants provided by Dehghan et al. and included 575,531 individuals of European ancestry (Additional file 1: Table S1) [13]. We analyzed only genetic variants that were independently associated (LD r2 < 0.001 within a 10,000 kb range) and had genome-wide significance (P < 5e–8) at the gene level for each inflammatory biomarker. In cases where the number of SNPs for inflammatory biomarkers was less than 3, we used a suggestive genome-wide P-value threshold (P < 5e–7) to identify a sufficient number of SNPs (at least 3) between the inflammatory biomarkers and HF [14, 15]. To address weak instrument bias in our instrumental variable analysis, we calculated the F-statistic for the selected SNPs as a test of weak instrument bias. A value greater than 10 was considered a strong instrument. To assess whether the selected SNPs were associated with other traits at a genome-wide significance level, we performed analyses using PhenoScanner (http://www.phenoscanner.medschl.cam.ac.uk/). We performed the main analyses after excluding potentially pleiotropic SNPs. The SNPs that were unavailable in the outcome dataset were searched for proxies in LDlink (R2 > 0.9).

## Outcomes

We included all type of HF patients in our study, encompassing those HF with preserved as well as reduced ejection fraction. The summary level data of HF obtained from the largest-scale GWAS meta-analysis involving participants of European ancestry (Additional file 1: Table S1) [16]. The meta-analysis included 26 cohorts (with a total of 29 distinct datasets) comprising 47,309 HF cases and 930,014 controls. HF was assessed through hospitalization, physician diagnosis or death records. Participants clinically diagnosed with HF were defined as cases, while those without HF were defined as controls. The clinical characteristics of the HF population was described in Additional file 1: Table S6. Among the heart failure patients, 27% were diagnosed with diabetes, compared to 6.5% in the control group. In the HF group, the proportion of male patients and the risk of occurrence for conditions such as hypertension (68.5% vs. 26.7%), chronic kidney disease (23.3% vs. 3.2%), myocardial infarction (39.2% vs. 4.7%), coronary heart disease (57.9% vs. 8.2%), and atrial fibrillation (36.6% vs. 3.1%) were all higher than those in the control group.

## Statistical analysis

When there is only a single SNP available for instrumental variable construction, the Wald ratio method is employed to derive MR estimates. In cases where multiple SNPs are available for instrumental variable construction, the inverse variance-weighted (IVW) method was used as the primary analysis because it provides the most precise and powerful estimates [17]. The Cochrane's Q statistic was used for the global test of heterogeneity in the IVW to evaluate heterogeneity among the genetic instruments. Considering the high correlation among inflammatory biomarkers, we performed multivariable MR to identify the most likely causally related inflammatory biomarkers. We used MR-BMA, a two-sample multivariable MR method, which can identify true causal risk factors even in the presence of a high correlation between candidate factors [18]. First, we conducted MR analyses using a weighted linear regression model for combinations of multiple inflammatory biomarkers similar to the IVW method and assessed the posterior probabilities of the causal relationship for each specific model within the Bayesian framework. Then, for each candidate inflammatory marker, we summed the posterior probabilities of all models that included the candidate inflammatory marker to calculate the marginal inclusion probability (MIP), which represents the probability of being a causal inflammatory marker for HF. Additionally, we computed the model-averaged causal effect estimates (MACE), representing the average causal effect of each inflammatory marker on HF. We calculated P-values for each inflammatory marker using permutation methods. For the assessment of the mediating effects of inflammatory markers on the association between SGLT-2 inhibitors and HF, we conducted a two-step MR analysis to evaluate the mediating effects of inflammatory biomarkers (Fig. 1). First, we estimated the effect of SGLT-2 inhibitors on 92 inflammatory markers using the univariable MR approach (β1). Second, we further selected those inflammatory markers showing a significant association with SGLT-2 inhibitor and estimated the effect of each inflammatory marker on HF (β2). The proportion of mediation by each inflammatory marker in the association between SGLT-2 inhibitors and HF was calculated as the product of β1 and β2 divided by the total effect of SGLT-2 inhibitors on HF. The 95% confidence interval (CI) for the mediation effect was calculated using the delta method [19].

## Sensitivity analyses

Sensitivity analyses were performed using the MR-Egger, MRPRESSO, weighted median, simple mode, and weighted mode methods. The MR-Egger method tests for the presence of horizontal pleiotropy, where a non-zero intercept value indicates the presence of horizontal pleiotropy and potential bias in the IVW estimate [20]. Additionally, the MR-PRESSO method was employed to assess the presence of horizontal pleiotropy by detecting possible outliers and recalculating the estimates after removing the outliers [21]. The weighted median method provides an unbiased causal estimate if at least 50% of the genetic instruments satisfy the three assumptions of MR [22]. The simple mode is a mode-based method that uses the causal effect estimates of single SNPs to form clusters and takes the largest SNP cluster for causal effect estimation [23]. The weighted mode method uses the same process but assigns weights to each SNP.

To address multiple hypothesis testing, we applied the Benjamini–Hochberg sequential p-value method to estimate the false discovery rate (FDR) adjusted p-values (q-values) [24]. A q-value less than or equal to 10% was considered significant [25]. A P-value < 0.05 for the effect of SGLT-2 inhibitors on HF was considered statistically significant. All MR analyses were conducted using the "TwoSampleMR," "MendelianRandomization," and "MRPRESSO" packages in R software (version 4.2.2). The code for the MR-BMA analysis can be found at the following URL: https://github.com/verena-zuber/demo_AMD.

## Results

### Impact of SGLT-2 Inhibition on HF

A total of 14 independent single nucleotide polymorphisms (SNPs) were selected as genetic instruments for SGLT-2 inhibition, with all SNPs having an F-statistic greater than 10 (Additional file 1: Table S2). We found a significant association between SGLT-2 inhibition and reduced risk of HF (0.42 [0.30–0.59], P < 0.0001), achieved by a 1-standard deviation decrease in HbA1c through SGLT-2 inhibition (Table 1). These results were supported by sensitivity analysis using MR-PRESSO. There was no evidence of heterogeneity among the genetic instruments (Q = 9.5282, P = 0.7320) and no detection of horizontal pleiotropy (Egger intercept = 0.0021, P = 0.8331).

**Table 1:** MR estimates of the effect of SGLT2 inhibition on heart failure

Method	Number of SNPs	OR (95% CI)	*P* value	*Q* statistic	*P*-heterogeneity	Egger-intercept	*P*-intercept
Inverse variance weighted	14	0.42(0.30–0.59)	0.0000	9.5282	0.7320		
MR egger	14	0.36(0.09–1.48)	0.1814	9.4818	0.6613	0.0021	0.8331
Simple mode	14	0.47(0.23–0.96)	0.0590				
Weighted median	14	0.46(0.29–0.72)	0.0007				
Weighted mode	14	0.44(0.23–0.83)	0.0242				
MR-PRESSO	14	0.42(0.31–0.56)	0.0000	10.6687	0.8170		

The heterogeneity test in the IVW methods was performed using Cochran’s *Q* statistic and the global test for the MR-PRESSO method. P < 0.05 was considered significant

*OR* Odds ratio, *CI* 95% confidence interval, *IVW* inverse–variance weighted, *P-heterogeneity* P value for heterogeneity test, *P-intercept* P value for the intercept of MR-Egger regression

### Impact of SGLT-2 inhibition on inflammatory biomarkers

**Fig. 2:**
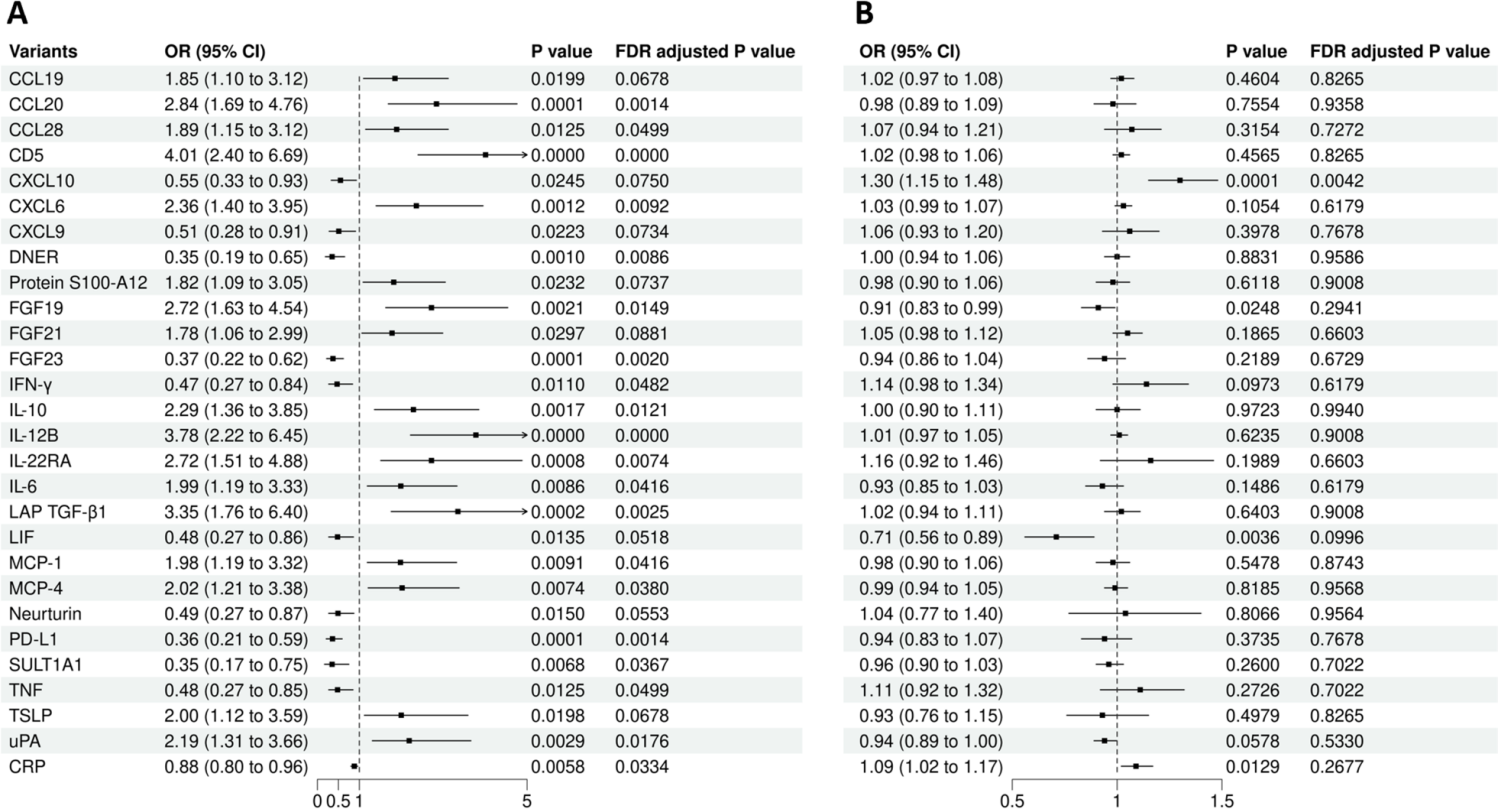
The forest plot of showing the effects of SGLT-2 inhibition on inflammation biomarkers and the effects of inflammation biomarkers on heart failure. **A** The effects of SGLT-2 inhibition on the remaining 28 selected inflammation biomarkers from 92 inflammation biomarkers, which were significantly associated with SGLT-2 inhibition. **B** The effects of the above 28 inflammation biomarkers on heart failure. *CI* confidence interval, *OR* odds ratio, *CCL19* C–C motif chemokine 19, *CCL20* C–C motif chemokine 20, *CCL28* C–C motif chemokine 28, *CXCL10* C-X-C motif chemokine 10, *CXCL6* C-X-C motif chemokine 6, *CXCL9* C-X-C motif chemokine 9, *DNER* Delta and Notch-like epidermal growth factor related receptor, *FGF19* Fibroblast growth factor 19, *FGF21* Fibroblast growth factor 21, *FGF23* Fibroblast growth factor 23, *IFN-γ* Interferon gamma, *IL − 10* Interleukin-10, *IL − 12B* Interleukin-12 subunit beta, *IL − 22RA* Interleukin-22 receptor subunit alpha-1, *IL − 6* Interleukin-6, *LAP TGF-β1* Latency associated peptide transforming growth factor beta 1, *LIF* Leukemia inhibitory factor, *MCP − 1* Monocyte chemoattractant protein-1, *MCP − 4* Monocyte chemoattractant protein-4, *PD − L1* Programmed cell death 1 ligand 1, *SULT1A1* Sulfotransferase 1A1, *TNF* Tumor necrosis factor, *TSLP* Thymic stromal lymphopoietin, *uPA* Urokinase type plasminogen activator, *CRP* C-reactive protein

We estimated the effects of SGLT-2 inhibition on 92 circulating inflammatory biomarkers and observed significant associations with 31 biomarkers (Table 2, Fig. 2A and Additional file 1: Table S3). We found that SGLT-2 inhibitors reduced the levels of CXCL10 (OR = 0.55 [95% CI 0.33–0.93], P = 0.0245, FDR-corrected P = 0.0750), leukemia inhibitory factor (LIF) (OR = 0.48 [95% CI 0.27–0.86], P = 0.0135, FDR-corrected P = 0.0518), and CRP (OR = 0.88 [95% CI 0.80–0.96], P = 0.0058, FDR-corrected P = 0.0334).

**Table 2:** Impact of SGLT-2 Inhibition on inflammatory biomarkers

Variants	OR (95% CI)	*P* value	FDR adjusted *P* value
CCL19	1.85 (1.10–3.12)	0.0199	0.0678
CCL20	2.84 (1.69–4.76)	0.0001	0.0014
CCL28	1.89 (1.15–3.12)	0.0125	0.0499
CD5	4.01 (2.40–6.69)	0.0000	0.0000
CXCL10	0.55 (0.33–0.93)	0.0245	0.0750
CXCL6	2.36 (1.40–3.95)	0.0012	0.0092
CXCL9	0.51 (0.28–0.91)	0.0223	0.0734
DNER	0.35 (0.19–0.65)	0.0010	0.0086
Protein S100-A12	1.82 (1.09–3.05)	0.0232	0.0737
FGF19	2.72 (1.63–4.54)	0.0021	0.0149
FGF21	1.78 (1.06–2.99)	0.0297	0.0881
FGF23	0.37 (0.22–0.62)	0.0001	0.0020
IFN-Y	0.47 (0.27–0.84)	0.0110	0.0482
IL-10	2.29 (1.36–3.85)	0.0017	0.0121
IL12B	3.78 (2.22–6.45)	0.0000	0.0000
IL-22RA	2.72 (1.51–4.88)	0.0008	0.0074
IL-6	1.99 (1.19–3.33)	0.0086	0.0416
LAP TGF-B1	3.35 (1.76–6.40)	0.0002	0.0025
LIF	0.48 (0.27–0.86)	0.0135	0.0518
MCP-1	1.98 (1.19–3.32)	0.0091	0.0416
MCP-4	2.02 (1.21–3.38)	0.0074	0.0380
Neurturin	0.49 (0.27–0.87)	0.0150	0.0553
PD-L1	0.36 (0.21–0.59)	0.0001	0.0014
SULT1A1	0.35 (0.17–0.75)	0.0068	0.0367
TNF	0.48 (0.27–0.85)	0.0125	0.0499
TSLP	2.00 (1.12–3.59)	0.0198	0.0678
uPA	2.19 (1.31–3.66)	0.0029	0.0176
CRP	0.88 (0.80–0.96)	0.0058	0.0334

*CI* confidence interval, *OR* odds ratio, *CCL19* C–C motif chemokine 19, *CCL20* C–C motif chemokine 20, *CCL28* C–C motif chemokine 28, *CXCL10* C-X-C motif chemokine 10, *CXCL6* C-X-C motif chemokine 6, *CXCL9* C-X-C motif chemokine 9, *DNER* Delta and Notch-like epidermal growth factor related receptor, *FGF19* Fibroblast growth factor 19, *FGF21* Fibroblast growth factor 21, *FGF23* Fibroblast growth factor 23, *IFN-γ* Interferon gamma, *IL-10* Interleukin-10, *IL-12B* Interleukin-12 subunit beta, *IL-22RA* Interleukin-22 receptor subunit alpha-1, *IL-6* Interleukin-6, *LAP TGF-β1* Latency associated peptide transforming growth factor beta 1, *LIF* Leukemia inhibitory factor, *MCP -1* Monocyte chemoattractant protein-1, *MCP-4* Monocyte chemoattractant protein-4, *PD-L1* Programmed cell death 1 ligand 1, *SULT1A1* Sulfotransferase 1A1, *TNF* Tumor necrosis factor, *TSLP* Thymic stromal lymphopoietin, *uPA* Urokinase type plasminogen activator, *CRP* C-reactive protein

### Impact of inflammatory biomarkers on HF

Among the 31 inflammatory biomarkers associated with SGLT-2 inhibitors, genetic instrumental could not be found for three biomarkers. Therefore, we listed the causal relationships between the remaining 28 inflammatory markers and HF (Additional file 1: Table S4, Fig. 2B). We found that two inflammatory biomarkers were significantly associated with HF. We observed a positive correlation between CXCL10 levels and HF (OR 1.30 [95% CI 1.15–1.48], P = 5.95e–5, corrected P = 0.0042). There was no evidence of heterogeneity among the genetic instruments (Q = 4.6105, P = 0.2026), and no detection of horizontal pleiotropy (Egger intercept = 0.0727, P = 0.5995). We also observed a negative correlation between LIF and HF (0.71 [0.56–0.89], P = 0.0036, corrected P = 0.0996).

### Results of multivariable analysis

In the MR-BMA analysis, we included CXCL10 and LIT, which were significantly associated with HF in the univariable MR analysis. The three models in MR-BMA analysis were respectively included for CXCL10, LIF, and both CXCL10 and LIF. These three models based on posterior probabilities are shown in Additional file 1: Table S5, while Table 3 displays the ranking of inflammatory biomarkers based on MIP. Additionally, the MACE values for each lipid are provided. The highest-ranking model included only CXCL10 (posterior probability = 0.797), which was also the strongest overall evidence for an inflammatory cytokine (MIP = 0.861, MACE = 0.224, FDR-corrected P = 0.0844).

**Table 3:** Prioritization of causal inflammation biomarkers of heart failure using the MR-BMA method

Inflammation biomarker	MIP	MACE	Empirical *P*-value	FDR adjusted *P* value
CXCL10	0.861	0.224	0.0422	0.0844
LIF	0.203	−0.018	0.9006	0.9006

Inflammation biomarkers ranked by the marginal inclusion probability in the MR-BMA analysis after model diagnostics. Empirical P-values were computed using 10,000 permutations. The prior probability and prior variance used in the MR-BMA analysis were 0.1 and 0.5

*MIP* marginal inclusion probability, *MACE* model-averaged causal effect, *CXCL10* C-X-C motif chemokine 10, *LIF Leukemia inhibitory factor*

### Mediation effects of inflammatory biomarkers

**Fig. 3:**
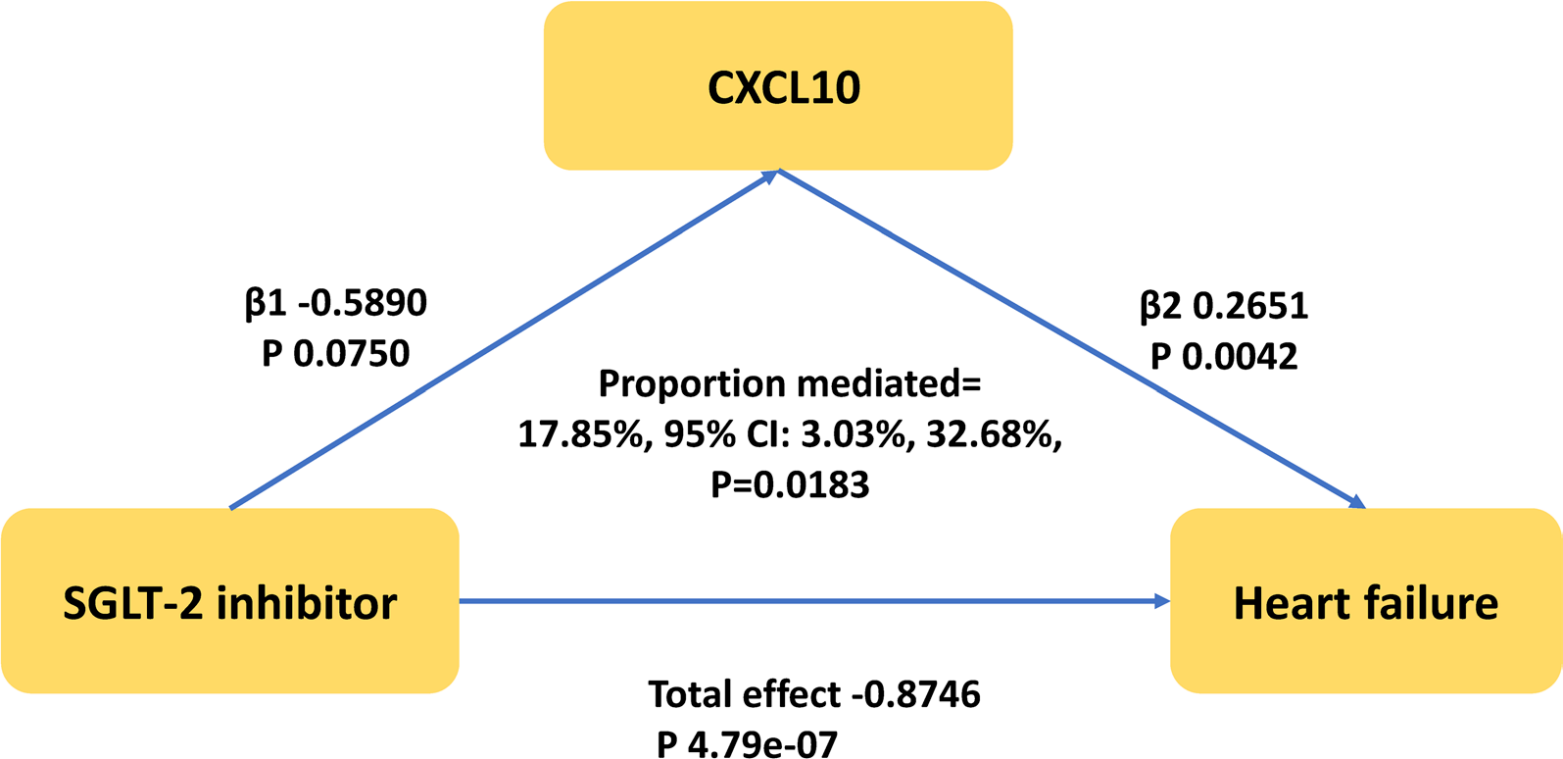
The CXCL10 mediated the causal effect of SGLT-2 inhibitor on heart failure. The β1 value between the SGLT-2 inhibitor and inflammation biomarker and the β2 value between inflammation biomarker and heart failure are MR estimates using the inverse–variance weighted method

Only two inflammatory biomarkers, CXCL10 and LIF, were found to be associated with both SGLT-2 inhibition and HF. Additionally, the highest-ranking model exclusively included CXCL10, which provided the strongest overall evidence for an inflammatory cytokine. Therefore, we reported only CXCL10 as biomarker in the mediation effect analysis. We observed that SGLT-2 inhibition had an indirect effect on the total effect of HF (OR 0.86 [95% CI 0.74–0.96], P = 0.0160) through CXCL10, with a mediation proportion of 17.85% (95% CI [3.03%–32.68%], P = 0.0183) (Central Illustration and Fig. 3).

## Discussion

In this study, we employed univariable and multivariable MR analyses and mediation analysis to evaluate the relationship between SGLT-2 inhibition, inflammatory biomarkers, and HF. Our findings suggest that SGLT-2 inhibitors may reduce the risk of heart failure (HF) by modulating inflammatory biomarkers, with CXCL10 potentially playing a mediating role—accounting for approximately 18% of the association between SGLT-2 inhibition and HF risk.

Previous clinical trials and meta-analyses have confirmed the effectiveness of SGLT-2 inhibitors in the prevention of HF [2] and the improvement in prognosis by SGLT-2 inhibitors is independent of the type of HF [3]. Our analysis encompassed all types of HF and demonstrated a significant negative correlation between SGLT-2 inhibitors and HF, aligning with clinical evidence. However, the exact mechanisms by which SGLT-2 inhibitors decrease the risk and prognosis of HF remain incompletely understood.

Emerging evidence indicates that SGLT-2 inhibitors possess the ability to modulate inflammatory responses [5]. Studies have exhibited that empagliflozin reduces pro-inflammatory biomarkers, including interleukin (IL)-6, tumor necrosis factor (TNF), monocyte chemoattractant protein (MCP)-1, interferon (IFN)-γ, P-selectin, and intercellular adhesion molecule (ICAM)-1 in the hearts of Zucker diabetic fatty rats [26, 27]. Canagliflozin has been shown to decrease serum leptin and IL-6 levels compared to glimepiride in patients with type 2 diabetes [28]. Empagliflozin treatment has also been associated with reductions in high-sensitivity C-reactive protein (hs-CRP) and myeloperoxidase, along with an increase in the anti-inflammatory biomarkers IL-10 [29]. Furthermore, a meta-analysis by Theofilis et al. reported reductions in IL-6, CRP, TNF-α, and MCP-1 levels with SGLT-2 inhibitors [6]. Consistent with these findings, our study revealed reduced levels of IFN-r, TNF, and CRP, as well as increased levels of IL-10, with SGLT-2 inhibitors. Additionally, utilizing a comprehensive pQTL dataset, our study identified significant effects of SGLT-2 inhibitors on CXCL9, CXCL10, DNER, FGF23, LIF, Neurturin, PD-L1, and SULT1A1. Our results were consistent with the study by Nakano et al., which showed that SGLT-2 inhibition directly suppressed tumor‐releasing CXCL10, suggesting a potential mechanism for the antitumor effects of SGLT-2 inhibitors in hepatocellular carcinoma [30].

Previous observational studies have provided insights into the relationship between inflammatory biomarkers, such as tumor necrosis factor (TNF) [31] and C-reactive protein [32], and HF. Recent MR studies have yielded contradictory findings regarding the role of inflammation in HF. Remmelzwaal et al. and Li et al. found no causal link between common inflammatory markers like CRP, TNF, IL-1, IL-6, and interleukins and HF [33, 34]. In contrast, Wei et al.’s larger study did find evidence of a causal relationship between certain inflammatory markers, including IL-2ra, IP-10, IL-6, and MIF, and HF [35]. In our study, we failed to observe a relationship between interleukins, growth factors, and HF. Furthermore, our analysis revealed a significant positive correlation between CRP and HF, though heterogeneity and pleiotropy among the genetic instruments were apparent. After outlier correction using the MR-PRESSO method, we found no causal relationship between CRP and HF. However, we did observe a significant impact of SGLT-2 inhibitors on CXCL10, and mediation analysis indicated that CXCL10 mediated the association between SGLT-2 inhibitors and HF.

CXCL10, a chemokine receptor closely associated with HF [36], exhibits elevated levels in symptomatic HF patients classified as New York Heart Association class II to IV. This chemokine, along with MIP-1α and CD40 ligand, appears to dominate in advanced-stage HF patients [37], highlighting the unique characteristics of inflammatory mediators in HF. Furthermore, T lymphocytes and the adaptive immune system have been implicated in the chronic inflammatory process of congestive HF. As a chemokine for T cells and a polarizing factor for pro-inflammatory phenotypes, CXCL10 plays a crucial role in recruiting Th1 cells to the heart in volume overload HF [38]. Therefore, the elevated levels of CXCL10 in HF patients and its role in promoting cardiac inflammation suggest its potential as a therapeutic target for HF. Previous clinical studies investigating anti-inflammatory agents as treatments for HF have yielded neutral results [39–42], indicating the need for further exploration of potential targets. Our study indicates that SGLT-2 inhibitors improve HF prognosis by inhibiting the inflammatory pathway, offering promising prospects for anti-inflammatory interventions in HF.

### Strengths and limitations of the study

This study is the first to investigate the relationship between SGLT-2 inhibition, inflammatory biomarkers, and HF using MR analysis. Nonetheless, our study has several limitations. Firstly, while simulating the genetic variations of SGLT-2 inhibitors may better reflect lifelong exposure, the effect sizes may not accurately represent the short-term effects. Therefore, MR analysis is more useful for examining potential directions of causality rather than quantifying effect sizes. However, the expected direction of effect can guide further exploration of therapeutic effects in clinical trials. Secondly, as our study was conducted using data from individuals of European ancestry, generalizing these results to other populations requires further investigation. Thirdly, despite the comprehensive range of inflammatory proteins included in our study, some inflammatory biomarkers were omitted, indicating the need for more comprehensive pQTL databases to explore additional potential targets. Lastly, the pathophysiology of HFpEF and HFrEF may differ, necessitating separate MR analyses for different populations. However, the limited availability of databases specific to certain types of HF makes achieving this purpose challenging. Nevertheless, previous studies have demonstrated that the benefits of SGLT-2 inhibitors in HF are independent of the HF type [3], and the relationship between inflammation and HF should be consistent across different types of HF [43, 44].

## Conclusion

This study provides genetic support for the relationship between SGLT-2 inhibition, inflammatory biomarkers, and HF. Specifically, CXCL10 appears to mediate the effect of SGLT-2 inhibitors on HF.

### Supplementary Information

**Additional file1: Table S1.** Detailed information for genome-wide association studies involved in the present Mendelian randomization study. **Table S2.** Instrumental variables for SGLT2 inhibition. **Table S3.** Mendelian Randomization analysis on the causal effect between SGLT-2 inhibitor and inflammatory biomarkers. **Table S4.** Mendelian Randomization analysis on the causal effect between inflammatory biomarkers and heart failure. **Table S5.** Ranking of models for heart failure according to their posterior probability after exclusion of outlying and influential variants in MR-BMA analysis. **Table S6.** Characteristics of participating studies.

## Data Availability

The GWAS Summary statistics used in this study were publicly accessed from the IEU OpenGWAS project (https://gwas.mrcieu.ac.uk/) and GWAS Catalog (https://www.ebi.ac.uk/gwas/home), the GTEx Portal (https://www.gtexportal.org/), the eQTLGen Consortium (https://eqtlgen.org/) and the DIAGRAM consortium (https://diagram-consortium.org/).

## Abbreviations

SGLT-2: Sodium-glucose cotransporter 2
MR: Mendelian Randomization
HbA1c: Glycated hemoglobin levels
ORs: Odds ratios
CI: Confidence interval
HF: Heart failure
SNP: Single nucleotide polymorphisms
IVW: Inverse variance-weighted (IVW)
CRP: C-reactive protein
CXCL9: C-X-C motif chemokine 9
CXCL10: C-X-C motif chemokine ligand 10
DNER: Delta and Notch-like epidermal growth factor-related receptor
FGF23: Fibroblast growth factor 23
IFN-r: Interferon gamma
LIF: Leukemia inhibitory factor
PD-L1: Programmed cell death 1 ligand 1
SULT1A1: Sulfotransferase 1A1
TNF: Tumor necrosis factor
CCL19: C–C motif chemokine 19
CCL20: C–C motif chemokine 20
CCL28: C–C motif chemokine 28
CXCL6: C-X-C motif chemokine 6
FGF19: Fibroblast growth factor 19
FGF21: Fibroblast growth factor 21
IL-10: Interleukin-10
IL-12B: Interleukin-12 subunit beta
IL-22RA: Interleukin-22 receptor subunit alpha-1
IL-6: Interleukin-6
LAP TGF-b1: Latency-associated peptide transforming growth factor beta 1
MCP-1: Monocyte chemoattractant protein-1
MCP-4: Monocyte chemoattractant protein-4
TSLP: Thymic stromal lymphopoietin
uPA: Urokinase-type plasminogen activator
FGF-19: Fibroblast growth factor 19
